# Mislocalization of death receptors correlates with cellular resistance to their cognate ligands in human breast cancer cells

**DOI:** 10.18632/oncotarget.542

**Published:** 2012-08-19

**Authors:** Jun-Jie Chen, H.-C. Jennifer Shen, Leslie A. Rivera Rosado, Yaqin Zhang, Xu Di, Baolin Zhang

**Affiliations:** ^1^ Division of Therapeutic Proteins, Office of Biotechnology Products, Center for Drug Evaluation and Research, Food and Drug Administration, Bethesda, Maryland, United States

**Keywords:** death receptors, cellular localization, targeted cancer therapy, drug resistance

## Abstract

Multiple clinical trials are ongoing to evaluate the potential antitumor activity of human TNF variants, Fas ligand (FasL), TNF-related apoptosis inducing ligand (TRAIL) and its agonistic antibodies. These drug products act through the death receptors (DRs) TNF receptor 1 (TNFR1), Fas/CD95, DR4 (TRAIL-R1) and/or DR5 (TRAIL-R2), respectively. Therefore, characterization of the level and localization of DR expression in cancer cells is important for DR-targeted therapy. In this study, we examined the subcellular distribution of the four DRs in a panel of 10 human breast cancer cell lines by western blots and flow cytometry and 50 human breast tumors by immunohistochemistry. Despite their total protein expressions, the DRs were found to be absent on the surface of some cell lines. Consistent with this result, all four DRs were found to be mostly expressed in the cytoplasm and/or the nucleus of primary breast tumors (n=50). We further determined the growth inhibition activity (GI50) of the death ligands, recombinant human TNFα, FasL and TRAIL, and found a correlation with the subcellular localization of the corresponding DRs. These results demonstrate an aberrant expression of the death receptors in breast cancer cells, and suggest that the lack of surface DRs appears to be predictive of tumor resistance to DR-targeted therapies.

## INTRODUCTION

The death receptors (DRs) TNFR1, Fas/CD95, DR4 and DR5 are attractive targets for cancer therapy due to their ability to induce apoptosis in various types of cancer cells, including breast cancer (1-4). These receptors are characterized by a death domain in the cytoplasmic tail which transduces signals from their cognate ligands such as TNFα, Fas ligand (FasL) and TNF-related apoptosis inducing ligand (TRAIL). Despite disappointing results from early clinical trials for recombinant TNFα, which showed severe toxicity after systemic exposure (5, 6), there is a continued effort to separate the unwanted toxicities from its potent apoptosis inducing activity by modifying TNF protein; for example, NGR-TNFα fusion protein (7) and TNFα-colloidal gold nanoparticles (8) are currently in clinical trails for treating human cancers. Fas/CD95 is also explored as a cancer therapeutic target (www.clinicaltrails.gov). A major move in the development of DR-targeted cancer therapy is the finding that TRAIL selectively induces apoptosis in cancer cells over most normal cells through activation of DR4 and/or DR5 expressed on target cells (9, 10). This unique property of TRAIL has generated considerable interest in clinically testing recombinant human TRAIL (rhTRAIL) and agnostic monoclonal antibodies against DR4 or DR5 (9, 11-13). However, tumor cells were often found to be sparsely sensitive to DR-mediated apoptosis or acquire resistance during therapy (14, 15). A better understanding of the resistance mechanisms could help identify molecular markers for predicting tumor response to DR-targeted therapies, thereby guiding the selection of patients for better treatment outcomes.

The classic mode of signal transduction for DRs involves activation of cell-surface DRs via binding of a cognate ligand, followed by the formation of a death-inducing signaling complex (DISC) at proximity of the inner layer of plasma membrane (1, 2). The key elements of a DISC include an adaptor protein TNFR-associated death-domain (TRADD) or Fas-associated death-domain (FADD), and pro-caspases 8 or 10. Within the DISC, caspase 8 or 10 becomes activated through auto-proteolysis that triggers activation of effector caspases (e.g. caspase 3), cleavage of many cellular substrates, and ultimately apoptotic cell death. Activation of TNFR1 or Fas can also recruit other components (e.g. RIP1 and TRAF2) into the DISC for activation of NFκ-B pathway (16). Increasing evidence indicates that the activation of DRs also triggers accelerated endocytosis of ligand-receptor complexes. For example, binding of TNFR1 or Fas induces the rapid clustering of the respective receptor, followed by internalization of the ligand-receptor complex *via* clathrin-coated pits formation (17-19). The internalized TNFR1 or Fas was shown to facilitate the assembly of secondary DISC complexes at intracellular endosomal compartments thereby amplifying the pro-apoptotic signal. Recent reports show that TRAIL receptors are also subject to regulation of endocytosis signals (20, 21). Our studies have shown that both DR4 and DR5 undergo constitutive or ligand-induced internalization in some breast cancer cell lines (14, 15, 22). Although the roles of DR4/DR5 endocytosis are just beginning to be understood, it may serve as a mechanism to terminate apoptosis signaling through TRAIL receptors (22).

We believe that understanding the relationship between differential expression and cellular localization of DRs will be beneficial in the development of biomarkers for predicting tumor response to the DR-targeted cancer therapies. In this study, we examined the cellular localization of the four DRs in breast cancer cell lines and primary breast tumors. We further compared DR cellular localization with cellular sensitivity to apoptosis induced by individual death ligands.

## RESULTS

### Distinct subcellular distribution of death receptors in breast cancer cell lines

**Figure 1:**
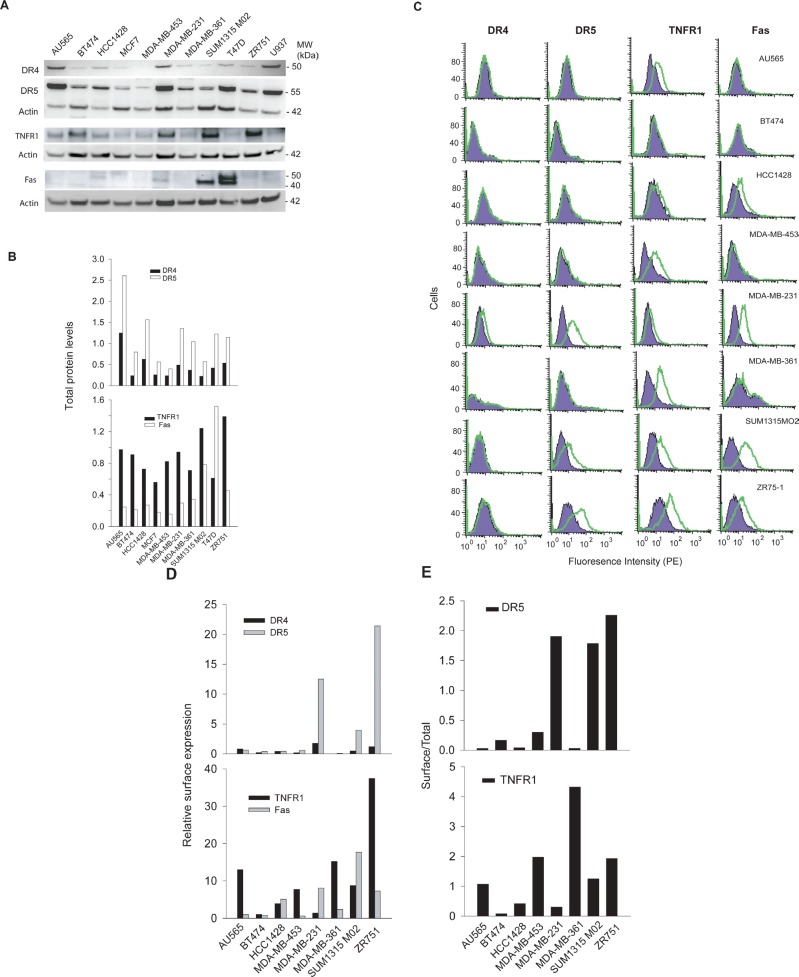
DR expression in breast cancer cell lines A, Protein expression levels of DR4, DR5, TNFR1, and Fas were analyzed by western blotting using antibodies specific to each receptor. B, The relative DR protein levels were estimated by densitometry analysis of the blots in A and normalized to the corresponding actin intensities. A monocyte cell line U937 was included as a control. C, DR expression on cell surface was determined by flow cytometry analysis after staining with PE-conjugated antibodies specific to each receptor (*open histograms*) or with isotype-matched control IgG (*shadowed histogram*). The presence of a DR on cell surface is indicated by the right-shift in fluorescence intensity compared to the control IgG-PE. D, Relative surface protein levels of DRs were estimated by the formula: [Mean (PE-DR) – Mean (PE-IgG)]/Mean (PE-IgG). E, Ratios of surface DR5 and TNFR1 expressions in 1D compared to their total protein levels as shown in 1B.

We examined the total protein expression levels of the death receptors (DRs) in a panel of ten randomly selected human breast cancer cell lines. Equal amounts of whole cell lysates were subjected to immunoblot analysis using antibodies specific to DR4, DR5, TNFR1, and Fas, respectively. The four receptors were found to be differentially expressed among the cell lines examined (Fig. 1A-B). For instance, the expression of DR4 and DR5 proteins was detected in most of the cell lines with a higher level in AU565 and MDA-MB-231 cells. TNFR1 was also widely expressed, whereas Fas was only detected in four cell lines including SUM1315 M02 and T47D.

Next, we investigated the DR expression on cell surface by flow cytometry using phycoerythrin (PE)-conjugated antibodies specific to each receptor. The presence of surface DR is indicated by a right-shift of the histogram peak relative to a control IgG-PE (Fig. 1C& 1D). The results showed distinct surface expression patterns for individual DRs. Specifically, we noted a differential surface positivity for DR4 (1/10), DR5 (3/10), TNFR1 (8/10), and Fas (4/10) among the ten cell lines. DR4 was only expressed on the surface of MDA-MB-231 cells while DR5 surface expression was detected for MDA-MB-231, SUM1315 M02 and ZR751 cells. Most cell lines expressed at least one DR on surface, except BT474 cells. MDA-MB-231 was the only cell line that expressed all four DRs on surface. Notably, the surface expression of a DR did not necessarily correlate with its total protein level in a specific cell line. In AU565 cells, for example, both DR4 and DR5 were deficient on cell surface while their total protein expressions were among the highest compared to other cell lines. We attempted to estimate the ratio between a surface DR and its total protein amount in a specific cell line. DR5 and TNFR1 were chosen for this analysis because their total protein expressions were detected in all the cell lines (Fig. 1A). The total protein levels were estimated by densitometry analysis of the blots in Fig. 1A, and the surface expressions were estimated by the right-shift values (mean fluorescence intensity) in Fig. 1C. Despite its total protein expression, little or no DR5 was present on surface of AU565, BT474, HCC1428, MDA-MB-453, and MDA-MB-361 cells (Fig. 1E). A different pattern was found for TNFR1, showing a higher frequency of surface expression (8 out of 10 cell lines).

**Figure 2:**
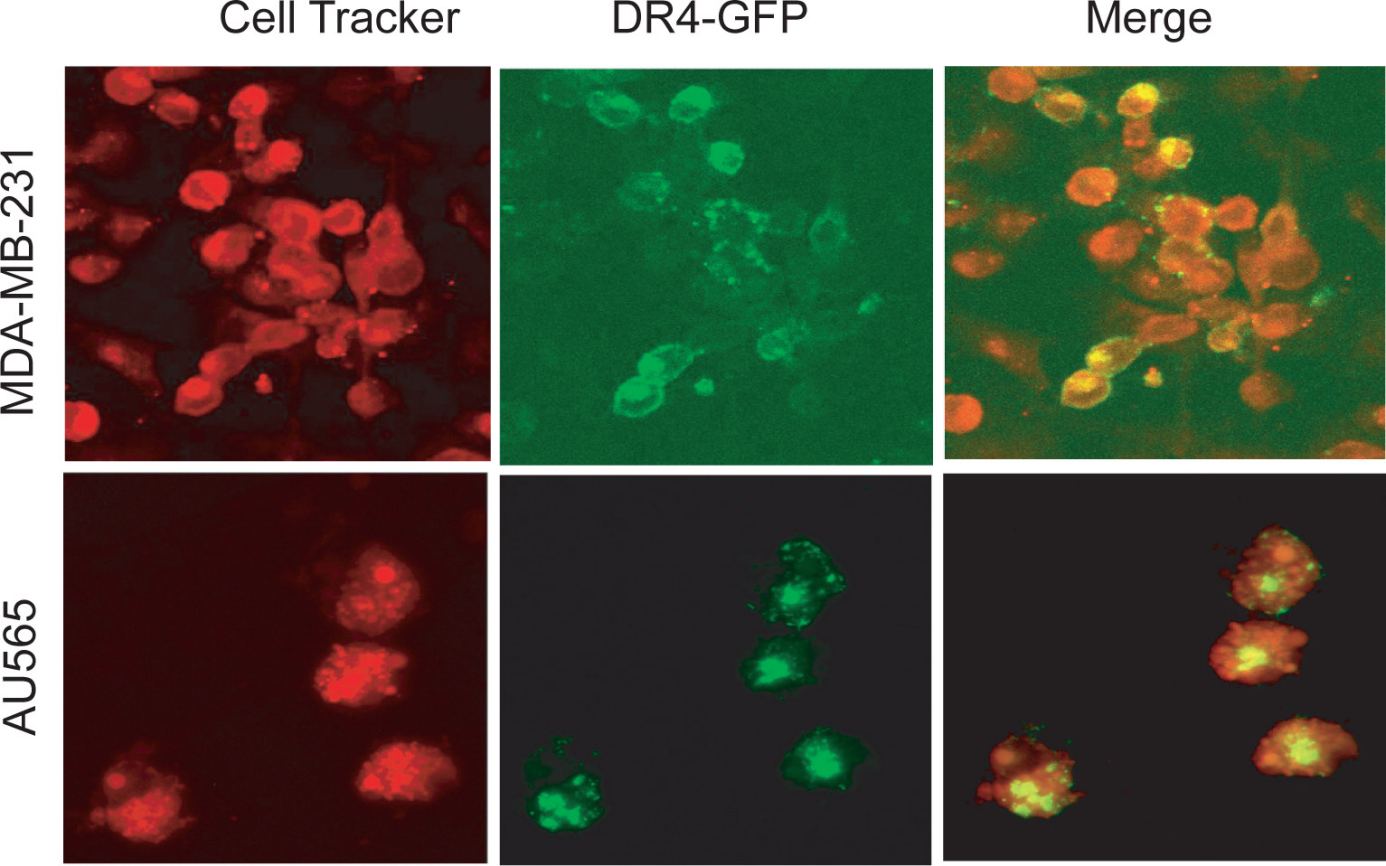
Confocal microscopy images showing the subcellular localization of ectopically expressed GFP-DR4 protein Cells were transiently transfected with GFP-DR4 expression plasmid (green), and counterstained with CellTracker Red (*red*). Images shown are representative of three independent experiments.

To further characterize the cellular localization of DRs, we transfected MDA-MB-231 and AU565 cells with a plasmid encoding GFP-DR4. As shown in Fig. 2, GFP-DR4 was clearly expressed on the surface of MDA-MB-231 cells although it was also detected in intracellular compartments. By contrast, GFP-DR4 was predominantly localized in the intracellular compartments with little or no surface expression in AU565 cells. These data are in agreement with the expression pattern observed for endogenous DR4 (Fig. 1C), demonstrating that certain breast cancer cell lines are defective in expressing DR(s) on cell surface.

### Surface deficiency of a death receptor is sufficient for rendering cellular resistance to apoptosis by its cognate ligand

**Figure 3:**
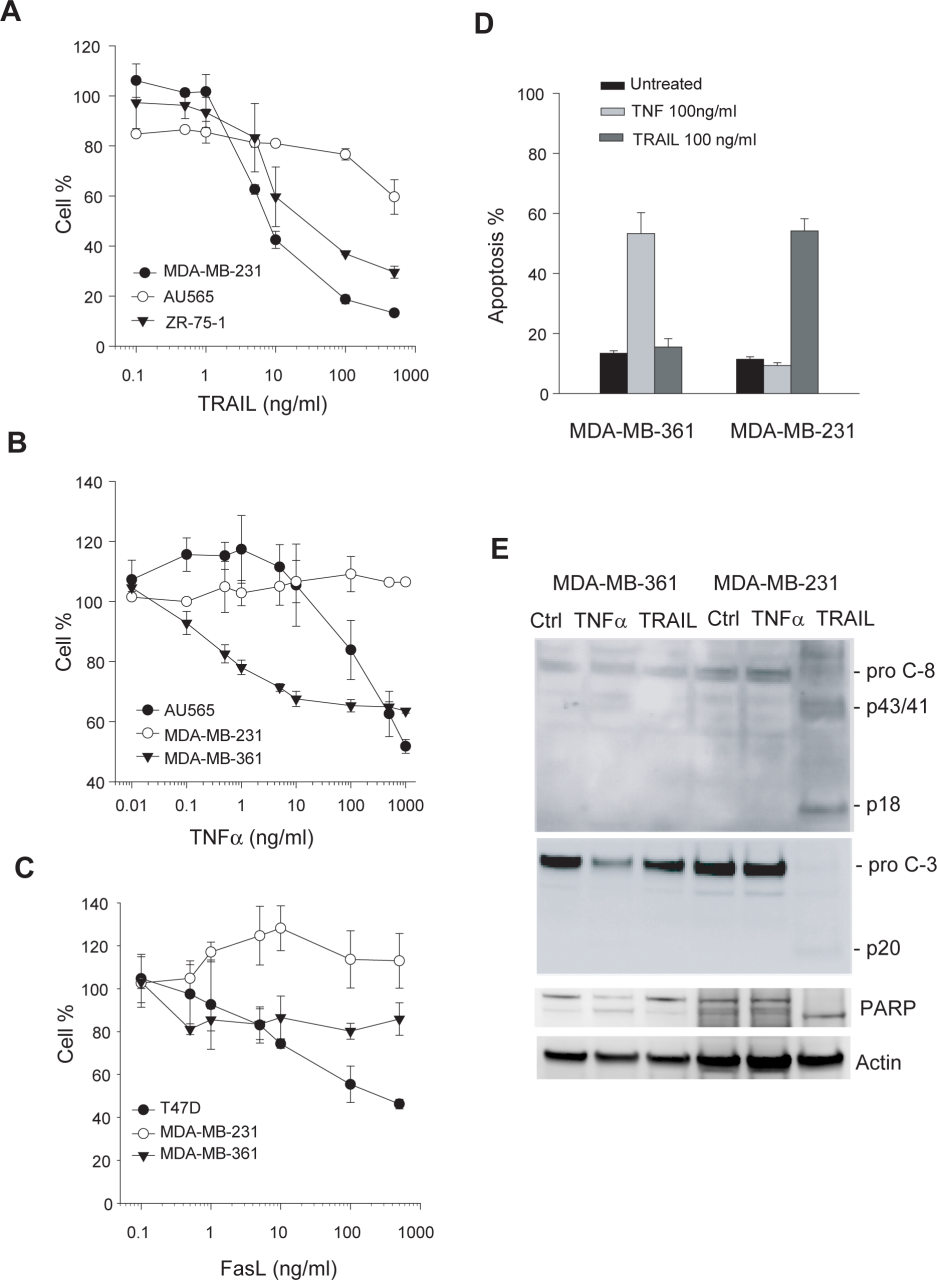
Cellular sensitivity to apoptosis induction by death ligands A-C, Representative dose-response curves in the indicated cell lines treated with increasing concentrations of rhTRAIL, rhTNFα, and rhFasL, respectively, at 37 C for 48 h. Cell viability was determined by a MTS assay (*see* Materials & Methods). GI50 values were derived from the response curves and summarized in Table 1. D-E, Apoptotic response to rhTRAIL or rhTNF in the indicated cell lines. Cells were treated with 100 ng/ml of rhTRAIL or 100 ng/ml of rhTNF at 37 C for 24 h, and analyzed by flow cytometry (D) and western blotting for caspase 3 and 8 (E). Activation of caspases was indicated by a decline in the pro-caspase forms, a simultaneous accumulation of cleaved fragments (p43/41 and p20), and the cleavage of a caspase substrate poly ADP ribose polymerase (PARP).

We asked if there is a relationship between the differential localization of DR expression and their ability to transduce a death signal from their corresponding ligands. To this end, we measured cell viability as a function of concentrations of rhTNF, rhTRAIL and rhFasL, respectively (Fig. 3A-C). The dose-response curves were fitted to derive GI50 values for individual cell lines (Table 1). The results show that most of these cell lines were resistant to the death ligands (GI50 > 500 ng/mL). Notably, the observed cellular resistance closely correlated with the absence of the corresponding DR on surface of target cells. On the other hand, the surface expression of a particular DR did not always correlate with cellular sensitivity to its cognate ligand. This was especially true for rhTNFα/TNFR1, where eight cell lines expressing surface TNFR1 were found to be resistant to rhTNFα-induced cell death. Among the ten cell lines tested, only MDA-MB-361 cells were sensitive to rhTNFα. This was confirmed by a direct measurement of apoptosis index and cleavage of caspase-8 and caspase-3 in the treated cells (Fig. 3D). Similarly, MDA-MB-231 cells were resistant to rhTNFα while remained highly sensitive to rhTRAIL. Overall, these results support the conclusion that lack of surface expression of a DR is sufficient for rendering cellular resistance to the cytotoxicity from its corresponding death ligand. Consistent with the notion that TRAIL could induce apoptosis through DR4 and/or DR5, TRAIL resistance occurred when both receptors were deficient on cell surface of target cells.

**Table 1:** Relationship between surface expression of the death receptors (DRs) and cellular sensitivity to their cognate ligands

Cell lines	DR Surface expression				IC50 (ng/mL) of death ligands		
	TNFR1	Fas/CD95	DR4	DR5	TNF	FasL	TRAIL
AU565	+		−	−	20-50	>500	>500
BT474	−		−	−	>500	>500	>500
HCC1428	+		−	−	>500	>500	>500
MCF7	+		+	+	>500	>500	>500
MDA-MB-453	+	−	−	−	>500	>500	>500
MDA-MB-231	+	+	+	+	>500	>500	1-5
MDA-MB-361	+	−	−	−	0.4-1	>500	>500
SUM1315 M02	+	−	−	+	>500	>500	>500
T47D	+		−	−	>500	3-5	>500
ZR751	+		−	+	>500	>500	7-8

### Death receptors are mostly expressed in cytoplasm and/or nucleus in primary breast tumor cells

**Figure 4:**
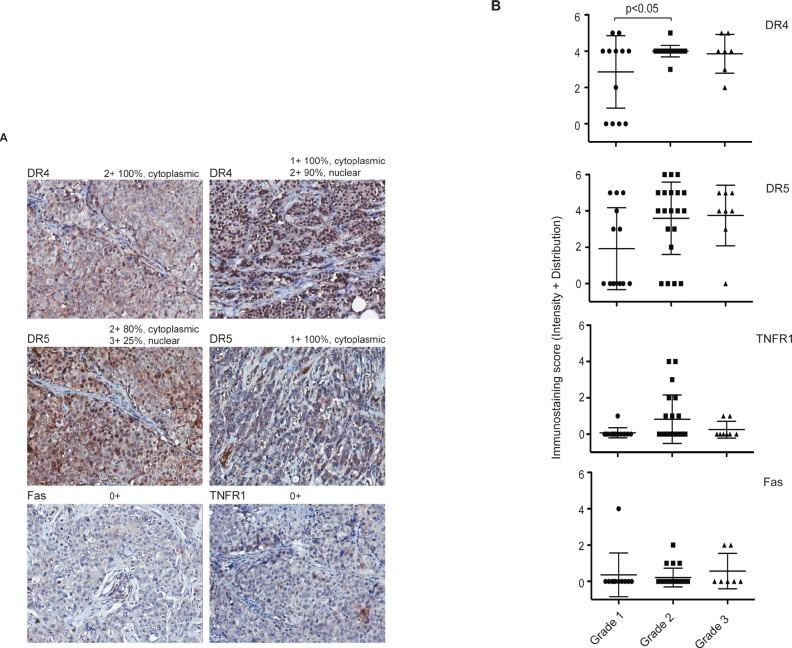
Immunohistochemical staining of DRs in primary breast tumors A. Representative images of DR4, DR5, TNFR1, and Fas immunohistochemical staining of human breast cancer tissue array (BR1005, US Biomax) with anti-DR4 (R&D Systems), anti-DR5 (ENZO), anti-Fas (Vector) or anti-TNFR1 (R&D). Magnification, × 200. B. Immuno-staining scores were plotted against tumor grades. Tissue samples were analyzed by a certified pathologist and given semi-quantitative scores based on staining intensity [0- no staining, 1- weak staining, 2- medium staining and 3- strong staining] and distribution [0 (<10% of cancer cells), 1 (10-40%), 2 (41-70%), and 3 (>70%)]. The final staining score (y-axis) was given by adding the intensity and the distribution for each sample. The average combined staining scores (intensity + distribution, ranging 0 to 6) are plotted (mean ± SD).

We next examined the expression of the four DRs in primary breast tumor tissues from 50 patients (n=50) by immunohistochemistry (IHC). Strikingly, specific staining of individual DR was noted in cytoplasm and/or nucleus of the cells (Fig. 4A). We also quantified the expression levels by combining the intensity and the distribution scores (*see* details in Materials & Methods). The scores for individual tumor samples are provided in Supplement I, and presented in Fig. 4B. All four DRs showed cytoplasmic and/or nuclear staining pattern through disease progression (Grade 1 to Grade 3). The immunostaining of DR4 and DR5 was noted to be stronger than those of TNFR1 and Fas. However, the expression levels of each DR did not differ significantly between tumor stages except a slight increase in DR4 from Grade 1 to Grade 2 (p>0.05) (Fig. 4B). Although the IHC staining did not exclude the possibility of surface expression, the staining patterns were generally consistent with the cell line data that indicate the mislocalization of DRs in breast cancer cells.

## DISCUSSION

The death receptor (DR)-targeted protein products have shown promise in early clinical trials for treating human cancers (7, 13, 23, 24). However, concerns about drug resistance in tumor cells are shifting the emphasis toward the identification of predictive biomarkers as well as effective combination therapies. In this study, we demonstrated the cell surface and intracellular localization of TNFR1, Fas, DR4 and DR5 in human breast cancer cells. Despite their total protein expression, these DRs were not expressed on cell surface of some cell lines. The surface deficiency of a particular DR correlated with cellular resistance to its corresponding death ligand. Strikingly, the expressions of the DRs in primary breast tumors were mostly localized to the cytoplasm and/or nucleus. Our results suggested that different localizations of DR expression (plasma membrane *versus* intracellular compartments) might be useful in identifying suitable patients for DR-targeted treatment.

Previous studies have demonstrated the expression of TNFR1, Fas, DR4, and DR5 at mRNA and total protein levels in a variety of cancer types. However, the results showed no direct link with the cellular sensitivity to the targeted therapies. Here, we examined the DR expressions in both whole cells and plasma membrane. In a ten cell line panel, the four DRs showed distinct patterns in their surface expression with positivity for DR4 (1/10), DR5 (3/10), TNFR1 (8/10), and Fas (4/10) (Fig. 1). In AU565 cells, for example, DR5 expression was among the highest in the panel but the receptor was not found on cell surface. The results were also consistent with our previous observation that DR4 was deficient at a higher frequency than DR5 in human cancer cells (15, 25). Importantly, the lack of surface expression of a specific DR correlated with the observed resistance to its corresponding death ligand (Table 1). On the other hand, the cell lines expressing surface DRs were not necessarily responsive to the ligands. This was especially true for TNFR1, which displayed a high percent of surface expression (8 out 10 cell lines) but a low responsive rate (2/8) to rhTNFα cytotoxicity (Fig. 3). We suspect that these cell lines may contain deficiency(ies) in the signaling components downstream of a specific DR, which may include downregulation of caspase-8 or upregulation of Bcl2, IAPs and other antiapoptotic proteins as found in many other cell types (25, 26). Nonetheless, the deficiency of cell surface DRs is sufficient for rendering cellular resistance to the DR-dependent therapies. Consistent with the cell line data (Fig. 1), the DRs were found to be mostly expressed in cytoplasm and/or nucleus in primary breast tumors (n=50). The diversity of resistance mechanisms suggests that tumors could be classified into four distinct groups: a) intact and functional DR pathways; b) DR deficiency on surface despite its total protein expression; c) deficiency in one or multiple intracellular signaling components (e.g. caspases, Bcl2, IAPs); and d) deficiency in both surface DR and its downstream signaling components. The combinational therapies should be designed to overcome or bypass the specific resistance mechanism(s) in a given tumor. In the cases of DR surface deficiency, one strategy would be to restore the surface expression of the DR on tumor cells. Further studies are underway to search for such drug candidates that could potentially enhance the clinical efficacy of the DR-dependent cancer therapies.

The mislocalization of DR4 and DR5 has been observed in other cell types, including CD4+ T cells (27) and cells of cervical neoplasia (28), melanoma (29), and non-small cell lung cancer (NSCLC) (30, 31). In these cases, both DR4 and DR5 were reported to be mainly expressed in cytoplasm and/or nucleus. However, little is known about the underlying molecular mechanisms. A recent study identified two nuclear localization signals in DR5 protein which mediates its nuclear localization through the nuclear import pathway by importin beta1 in Hela and HepG2 cells (32). The overexpression of DRs in cytoplasm may reflect receptor-ligand internalization, a rapid process occurring after ligand binding. Numerous studies have shown that TNFR1 and Fas undergo rapid internalization in response to ligation (17-19). TRAIL receptors, DR4 and DR5, were also shown to follow a similar mode of action (14, 15, 20-22). In a tumor setting, this might be triggered by soluble cytokines (TNFα, FasL or TRAIL) in the tumor microenvironment (33). The signaling events may involve clathrin-dependent endocytosis or other uncharacterized mechanisms (15, 20, 21). However, we can not exclude the possibility that intracellular localization of DRs may be newly synthesized molecules within the endoplasmic reticulum or Golgi that have yet to be processed and inserted properly into the plasma membranes. In any event, the trapped DR in cytoplasm inevitably reduces its accessibility to incoming ligands, thereby making the cells resistant to the targeted therapies.

Numerous efforts have been made to identify chemotherapeutic agents that can be used in combination with the DR-targeted agents to overcome or bypass the resistance mechanisms (6, 34, 35). For instance, flavopiridol (34) and actinomycin D (6) have been shown to potentiate the cytotoxicity of rhTNFα in cells of lymphoma and prostate carcinoma. Consistent with these results, both agents are found to enhance rhTNFα induced apoptosis in MDA-MB-231 and BT474 breast cancer cell lines (Supplement I). However, there was no apparent effect on surface expression of TNFR1 in the treated cells. As previously proposed (34), the synergy effect may be a result of inhibition of TNFα-induced expression of inhibitors of caspases. These data are in line with other reports showing the diversity of resistance mechanisms which highlights the necessity for the identification of combinational therapies to target the specific mechanism(s) of drug resistance in individual cancer patients.

Of clinical relevance, the mislocalization of DRs was also observed in primary breast tumor cells. Further studies should aim at investigating whether different patterns of immunostaining (cell surface *versus* cytoplasm or nucleus) might help to select patients for different approaches for DR-targeted treatment. In addition to breast cancer, the DR-targeted protein products are being clinically evaluated for treating many other malignancy types. Therefore, it will be also of importance to determine the cellular localization of DRs in other tumors.

## MATERIALS AND METHODS

### Cell lines

Human breast cancer cell lines including AU565, BT474, HCC1428, MCF7, MDA-MB-453, MDA-MB-361, MDA-MB-231, T47D, and ZR751 were obtained from the American Type Culture Collection (Manassas, VA), and SUM1315-MO2 was from Asterand (Detroit, MI), and grown per supplier's instructions. All cell lines were routinely tested for mycoplasma contamination, and were cultured no longer than 3 months before replacing with freshly thawed stocks.

### Western blotting

Immunoblotting analysis was performed as described (22). Cell lines were cultured under normal growth conditions, harvested, and lysed in 50 mM Tris-HCl buffer containing 2% SDS. Protein concentrations were determined using the BCA protein assay (Pierce, Rockford, IL). Equal amounts of cell lysates were resolved by electrophoresis using 4-12% NuPAGE Bis-Tris gels (Invitrogen) and transferred to PVDF membranes (Millipore). Primary antibodies were used at an appropriate dilution of 1:100 to 1:2000. Monoclonal anti-DR4 (Clone 32A242) and polyclonal anti-DR5 antibodies were purchased from Imgenex (San Diego, CA). Antibodies against human Fas, caspase-3, caspase-8 were from Cell Signalling Technology (Danvers, MA), and anti-actin from Santa Cruz Biotechnology (Santa Cruz, CA). When necessary, the membranes were stripped by Restore western blot stripping buffer (Pierce) and reprobed with appropriate antibodies. Immunocomplexes were visualized by chemiluminescence using ECL reagent (Santa Cruz).

### Flow cytometric analysis for cell surface expression of death receptors

FACS analysis of cell surface expression of TNFR1, Fas, DR4 and DR5 was performed using the phycoerythrin (PE) -conjugated antibodies as described previously (22). The phycoerythrin (PE)-conjugated forms of anti-TNFR1 (IgG1, FAB225P), anti-Fas (IgG1, FAB142F), anti-DR4 (IgG1, FAB347P), and anti-DR5 (IgG2b, FAB6311P) antibodies as well as IgG1 and IgG2b controls were purchased from R & D Systems (Minneapolis, MN). In brief, cells at 70-80% confluence were harvested by incubation with an enzyme-free cell-dissociation Buffer (Invitrogen) and washed twice in culture medium. Cells (~5 × 10^5^) were incubated in blocking solution (phosphate buffered saline containing 5 % goat serum and 1% bovine serum albumin) for 20 min on ice. Afterwards, cells (30 μL) were incubated with 10 μg/mL of PE-conjugated antibodies to the death receptors for 45 min at 4 °C in the dark. Duplicate samples were incubated with the respective IgG1-PE or IgG2b-PE as negative controls. Cells were then washed twice with PBS and resuspended in 0.5 mL PBS for flow cytometry analysis using a FACS Calibur (BD Biosciences).

### Immunofluorescence microscopy

Cells were cultured on glass chamber slides at 70-80% confluency and transiently transfected with a plasmid for expression of GFP-DR4 (15). After 24 h post-transfection, cells were stained with CellTracker Red (Invitrogen) and fixed in 3% paraformaldehyde for 30 min in PBS, pH 7.4. Finally, the slides were detached from the medium chamber and mounted with Antifade reagent (Invitrogen). Confocal microscopy acquisitions were performed on a Zeiss LAM 5 PASCAL confocal laser scanning microscope. All images are representative of 3-5 independent experiments.

### Cytotoxicity assays

Cell viability was assessed using a MTS assay (Promega One Solution Cell Proliferation Assay (G3580). Briefly, cells were seeded in 96-well plates at plating densities ranging from 5000 to 10000 cells/well depending on the doubling time of individual cell lines. After 24 h incubation, some of the wells were processed to determine a time zero density. To the rest of the plates, individual death ligand (rhTNF, rhFasL or rhTRAIL) was added at seven different doses (0.1, 0.5, 1, 5, 10, 100, and 500 ng/mL). Plates were incubated for another 24 h, then stained with CellTiter One Solution Reagent, and measured for absorbance at 490 nm. Growth inhibition of 50 % (GI50), which is the drug concentration resulting in a 50% reduction in the net protein increase (as measured by MTS staining) of control cells during the drug incubation, is calculated from [(Ti-Tz)/(C-Tz)] × 100 = 50 using absorbance at 515 nm at time zero (Tz), in the absence of death ligand (C), and in the presence of death ligand (Ti). Recombinant human TNF-α/TNFSF1A (hTNF, 166 amino acids corresponding to Val77-Leu233 of human TNF-α), human Fas ligand/TNFSF6 (hFasL, 148 amino acids corresponding to Pro134-Leu281 of human FasL), and human TNF-related apoptosis inducing ligand/TNFSF10 (rhTRAIL, 168 amino acids corresponding to Val114 – Gly281 of human TRAIL), which were expressed by E. *coli* or CHO cells and purified as homotrimeric proteins, were from R & D systems (Minneapolis, MN).

For apoptosis analysis, cells were grown on 6-well plates to 70-80% confluence and treated with individual death ligand at the indicated concentrations. At the selected time points, cells were collected and analyzed by flow cytometry after staining with annexin V-FITC (Pharmingen) and propidium iodide (PI) (36). Apoptotic cells were characterized by positive staining of annexin V-FITC and/or PI. All experiments were performed in triplicate and at least 3 repetitive experiments were performed for each result.

### Immunohistochemistry

The expression of death receptor proteins in primary breast tumors was assessed using the commercial breast cancer tissue microarrays (TMAs) from US Biomax (Rockville, MD). Informed consent has been obtained and kept on file at the tissue banks. The donor's identity is anonymity and all tissues and data are labeled using only ID Codes. All the tissue samples were preserved in 10% phosphate buffered formalin (pH 7.4), embedded in paraffin, processed into sections. Array sections (4 μm thick) were mounted on the positive charged super plus glass slide. Each individual core was 1.5 mm in diameter and spaced 0.25 mm. Pathology diagnosis of tissues in TMAs was provided by the vendor, and confirmed at our laboratory by the microscopic evaluation of the histopathology of the cores by a board certified pathologist.

For immunohistochemistry (IHC) analysis, tissue sections were deparaffinized in xylene, and rehydrated in gradients of alcohol and water. Endogenous peroxidase activity was blocked by incubating slides in 3% hydrogen peroxide at room temperature for 5 minutes. Antigen retrieval was performed in the Target Retrieval solution (Dako Cytomation) for 30 minutes in microwave oven. To reduce non-specific staining, slides were washed in phosphate buffered saline with Tween-20, followed by incubation in 2.5% normal horse blocking serum for 30 min. Blocked sections were incubated in primary anti-DR antibody for 1 hour at room temperature. Antibodies for immunohistochemistry were: anti-DR4 (#AF347, R&D Systems), anti-DR5 (#ALX-210-743-C200, Enzo Life Sciences) anti-Fas/CD95 (#VP-F702, Vector Lab) and anti-TNFR1 (#MAB225, R&D Systems). After washing three times, slides were incubated for 30 minutes with ImmPRESS reagent (Vector Laboratories) followed by incubation with the peroxidase substrate DAB solution (DAKO Cytomation) until desired stain intensity develops. Slides were counterstained with Hematoxylin and mounted with permanent mounting medium. Evaluation of the IHC was performed by a board certified pathologist.

The IHC score was determined by combining the intensity and distribution scores (percentage of the positivity in the tumor tissues) scores (37). The staining intensity was based on a 4-point system: 0 (no staining), 1 (weak), 2 (moderate), and 3 (strong). The distribution score was assessed as follows: 0 = less than 10% of the cancer cells stained on the sections; 1 = 10% to 40%; 2 = 40% to 70%; and 3 = more than 70%. The final score was reported by the sum of the intensity and the distribution scores, yielding a score range between 0 and 6.

### Statistical analysis

Statistical analyses were performed with Prism (GraphPad). All cell-based studies were done in triplicate. Data were presented as mean + SD. Statistical significance was defined as *P* < 0.05.

## Supplementary Figures


