# Supplementary material for: Mislocalization of death receptors correlates with cellular resistance to their cognate ligands in human breast cancer cells
Source: Oncotarget. 2012 Aug 19;3(8):833–42. doi: 10.18632/oncotarget.542 (PMC3478460; doi:10.18632/oncotarget.542)
Supplement: Supplementary file 1 [file oncotarget-08-833-s001.docx]

Mislocalization of death receptors correlates with cellular resistance to their cognate ligands in human breast cancer cells – Chen etal
